# Supplementary figures and images for: Understanding the Effect of Different Glucose Concentrations in the Oligotrophic Bacterium Bacillus subtilis BS-G1 through Transcriptomics Analysis
Source: Microorganisms. 2023 Sep 26;11(10):2401. doi: 10.3390/microorganisms11102401 (PMC10609351; doi:10.3390/microorganisms11102401)

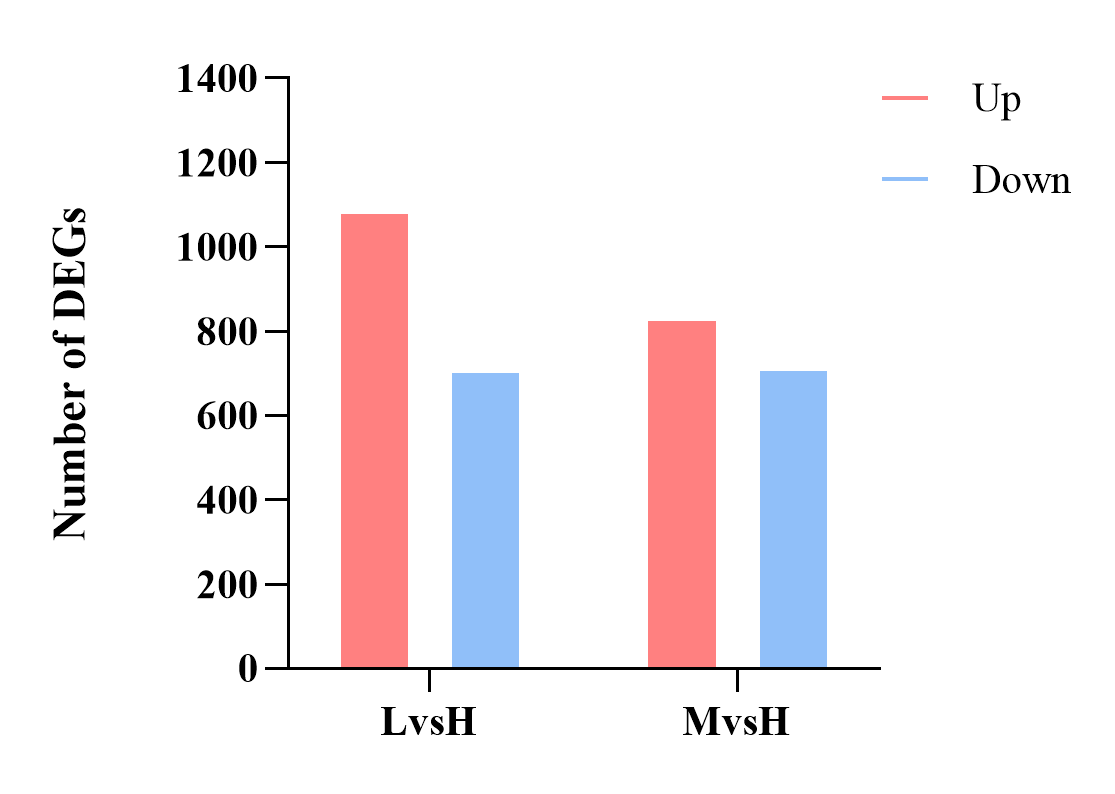

Supplement: Supplementary file 1 [file microorganisms-11-02401-s001.zip › Figure S1. The statistic of DEGs number.tif]

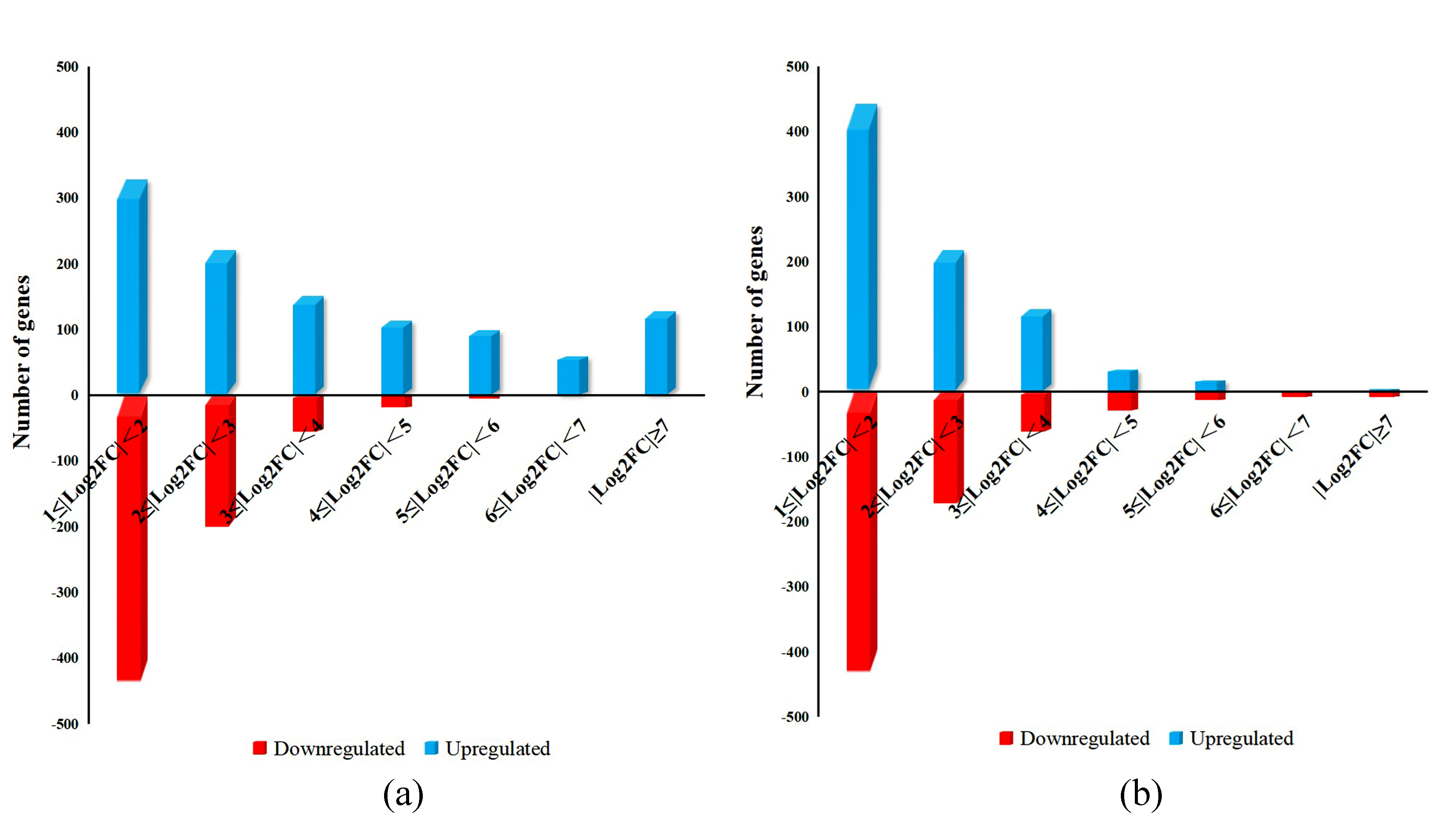

Supplement: Supplementary file 1 [file microorganisms-11-02401-s001.zip › Figure S2. Fold change distribution of DEG present in samples with different treatments.tif]

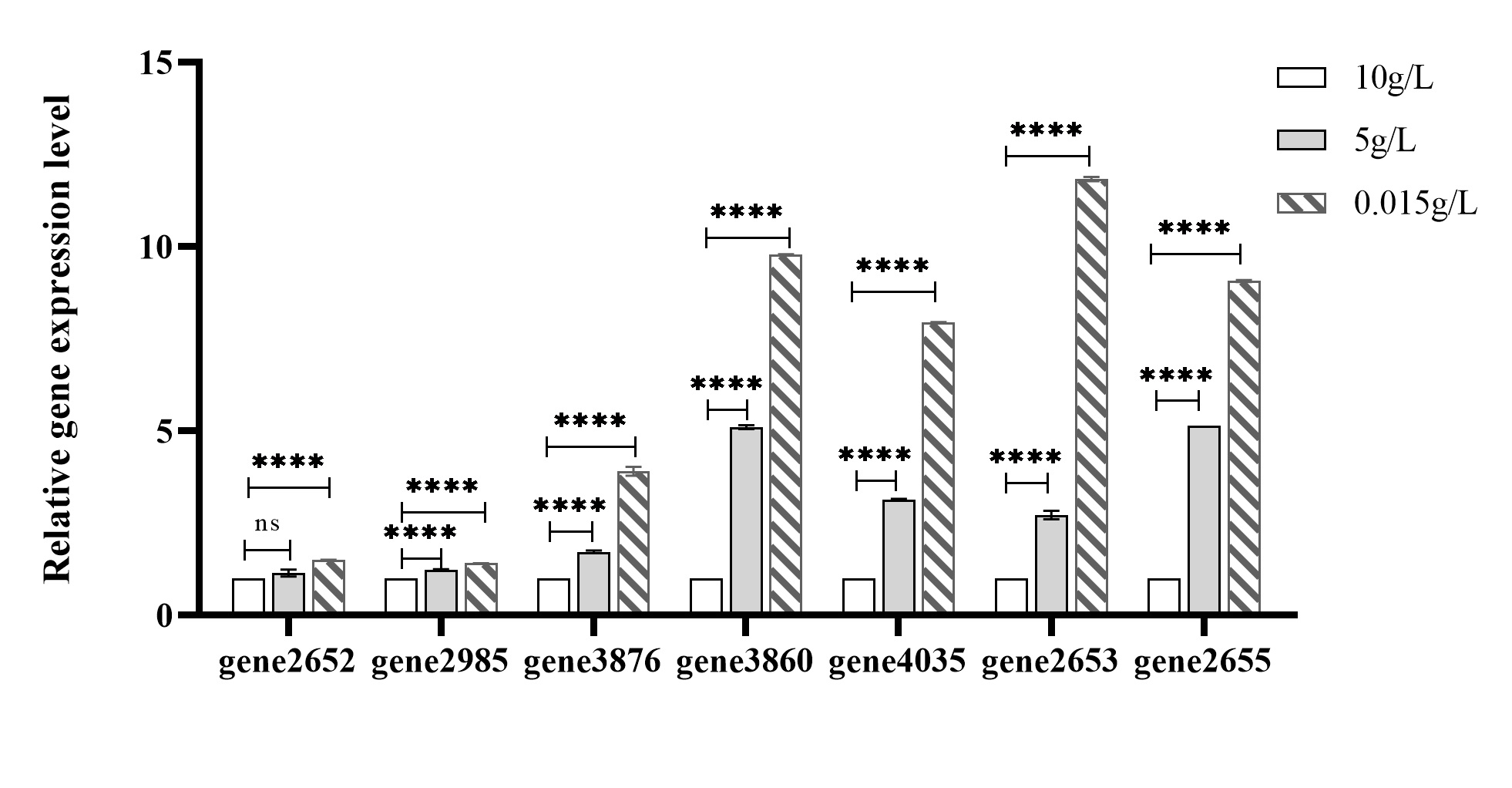

Supplement: Supplementary file 1 [file microorganisms-11-02401-s001.zip › Figure S3. The relative mRNA expression of genes determined by qRT-PCR.tif]
